# Ocular brachytherapy dosimetry for ${}^{103}\text{Pd}$ and ${}^{125}I$ in the presence of gold nanoparticles: a Monte Carlo study

**DOI:** 10.1120/jacmp.v17i3.5945

**Published:** 2016-05-08

**Authors:** Somayeh Asadi, Mehdi Vaez‐zadeh, Mohammad Vahidian, Mahdieh Marghchouei, S. Farhad Masoudi

**Affiliations:** ^1^ Department of Physics K.N. Toosi University of Technology Tehran Iran

**Keywords:** brachytherapy, choroidal melanoma, MCNP5, gold nanoparticles, ${}^{103}\text{Pd}$ and ${}^{125}I$

## Abstract

The aim of the present Monte Carlo study is to evaluate the variation of energy deposition in healthy tissues in the human eye which is irradiated by brachytherapy sources in comparison with the resultant dose increase in the gold nanoparticle (GNP)‐loaded choroidal melanoma. The effects of these nanoparticles on normal tissues are compared between ${}^{103}\text{Pd}$ and ${}^{125}I$ as two ophthalmic brachytherapy sources. Dose distribution in the tumor and healthy tissues has been taken into account for both brachytherapy sources. Also, in certain points of the eye, the ratio of the absorbed dose by the normal tissue in the presence of GNPs to the absorbed dose by the same point in the absence of GNPs has been calculated. In addition, differences of the absorbed dose in the tumor observed in the comparison of simple water phantom and actual simulated human eye in presence of GNPs are also a matter of interest that have been considered in the present work. The difference between the eye globe and the water phantom is more obvious for ${}^{125}I$ than that of the ${}^{103}\text{Pd}$ when the ophthalmic dosimetry is done in the presence of GNPs. Whenever these nanoparticles are utilized in enhancing the absorbed dose by the tumor, the use of ${}^{125}I$ brachytherapy source will greatly amplify the amount of dose enhancement factor (DEF) in the tumor site without inflicting much damage to healthy organs, when compared to the ${}^{103}\text{Pd}$ source. For instance, in the concentration of 30 mg GNPs, the difference amongst the calculated DEF for ${}^{125}I$ between these phantoms is 5.3%, while it is 2.45% for ${}^{103}\text{Pd}$. Furthermore, in Monte Carlo studies of eye brachytherapy, more precise definition of the eye phantom instead of a water phantom will become increasingly important when we use ${}^{125}I$ as opposed to ${}^{103}\text{Pd}$.

PACS number(s): 87.53.Jw, 87.85.Rs, 87.10.Rt

## I. INTRODUCTION

Uveal melanoma is one of the primary ocular cancerous tumors which arises within the eyeball in the uvea involving the iris, ciliary body, or choroid. Although this kind of cancer is rare, it is the most common eye cancer in people who are middle‐aged or older.^1^, ^2^, ^3^, ^4^ Regarding the size and location of the tumor and also the rate of its progress, treatment of the choroidal melanoma is managed. Enucleation, local resection, and radiotherapy are the most common therapeutic processes for the treatment of choroidal melanoma.^5^, ^6^, ^7^

Considering the sensitive tissues which the eye is involved in, ocular tumors present a therapeutic challenge.^(8)^ Although the radiation is effective therapeutically, it can be harmful to healthy tissues. Plaque brachytherapy is the most widely used treatment, which aims to transfer the maximum amount of dose to the tumor while preventing dose absorption by normal tissue.^9^, ^10^ However, in the period of treatment both the tumor and the proximal normal tissues obey a certain pattern in radiation absorption; hence, in this therapeutic procedure minimizing the absorbed dose by the normal tissue is still one of the major concerns. Applying gold nanoparticles (GNPs) as radiation dose enhancers in combination with brachytherapy can be an effective method to reduce the radiation effects on healthy nearby tissues.

In the *in vivo* study by Hainfeld et al.,^11^, ^12^ on the use of GNPs in mice which were irradiated by X‐ray photons, the results show that presence of GNPs in the tumor will cause more radiation dose in the cancerous cells than in those of the healthy tissues. In the *in vitro* study by Kong et al.,^(13)^ on the application of GNPs to enhance radiation cytotoxicity, the results show that radiotherapy killed more cancerous cells in the presence of GNPs than in the absence of these nanoparticles. Subsequently, GNP radiosensitization has been observed in more controlled *in vitro* irradiation of cells and plasmid DNA.^14^, ^15^, ^16^

The idea of using gold nanoparticles in cancer therapy as a radiosensitizer is not a new one and several Monte Carlo *in vitro* and *in vivo* studies on the application of nanotechnology‐based cancer therapy have been performed.^17^, ^18^, ^19^, ^20^ However, only a handful of studies, such as our previous one,^(21)^ have yet been conducted to investigate the effects of these nanoparticles on human eye tumors such as choroidal melanoma. If the choroidal melanoma tumor could be loaded with these nanoparticles in proper concentrations and dimensions, this would lead to a higher absorbed dose by the tumor during a shorter time. Since the eye is an extremely sensitive organ, the reduction of the period of treatment would decrease the absorbed dose by normal cells, resulting in a major reduction in the damage inflicted.

Regarding the radiosensitizing properties of gold nanoparticles, dose enhancement in the tumor is expected when the GNP‐loaded choroidal melanoma is locally irradiated with brachytherapy sources. However, considering the healthy tissues and the different seed models which are used in the eye plaque therapy, the variation of energy deposition in normal tissues in comparison with the resultant dose increase in the tumor by diverse sources is of outmost importance in the investigation of GNP effects on ophthalmic brachytherapy dosimetry. Some studies have been carried out through making a dosimetry comparison between ${}^{103}\text{Pd}$ and ${}^{125}I$.^3^, ^22^ These studies reported higher absorbed dose by the tumor for ${}^{103}\text{Pd}$ versus ${}^{125}I$ for an equivalent radiation time. However, the focus of the present study is to know what changes will occur in absorbed dose by the healthy tissues compared to the dose increase in the tumor when the nanoparticle‐induced tumor is irradiated with ${}^{125}I$ and ${}^{103}\text{Pd}$.

Here, a rigorous comparison between low‐energy photon sources ${}^{103}\text{Pd}$ and ${}^{125}I$ within COMS eye plaques was provided in the study of the effects of GNPs in ophthalmic brachytherapy. We also studied the difference between water phantom and human eye globe in the presence and absence of these nanoparticles for both the mentioned sources. The mean absorbed dose to the apex of the tumor, as well as to other critical points in both water and eye phantoms, were calculated in order to evaluate the effects of both sources and identify the most efficient source with the least amount of induced cell damage to critical points and healthy tissue in the presence of GNPs.

**Figure 1:**
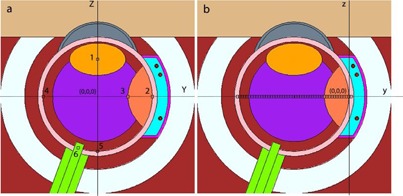
This longitudinal cross‐sectional diagram of the simulated human eye represents a simplified eye model. In the left pane (a), the origin of the eye coordinate system is incident upon the center of the eye phantom. The voxels (numbered 1 to 6) indicate the lens, sclera, tumor apex, opposite side, macula, and optic nerve, respectively. In the right pane (b), the origin of the plaque coordinate is at the interior shell of the sclera.

To this end, the eye globe was simulated precisely by considering different parts of the eye and their components. Here, both water and eye phantoms were simulated with MCNP5 code in which the water and eye phantoms were nearly identical owing to the fact that water was taken as the composition of the simulated eye globe, albeit by considering accurate geometry of the noted organ (Fig. 1).

The geometric characteristic of this phantom is in accordance with the eye of an adult. GNPs 50 nm in diameter were chosen, since they portray maximum uptake in mammalian cells.^(23)^ Dosimetric characteristics of a single source for ${}^{125}I$ and ${}^{103}\text{Pd}$ were utilized to validate the accuracy of the Monte‐Carlo simulation technique. Fully‐loaded 16 mm COMS standard eye‐plaque with ${}^{125}I$ source (model 6711, GE Healthcare/Oncura, Arlington Heights, IL), and ${}^{103}\text{Pd}$ source (model 200, TheraSeed, Theragenics Corp., Buford, GA) were the focus of this study.

## II. MATERIALS AND METHODS

The present Monte Carlo simulations are carried out using MCNP5 code.^(24)^ The mentioned code benefits from a three‐dimensional heterogeneous geometry for both photons and electrons situated within the energy range of 1 KeV to 1 GeV; moreover, the libraries incorporated within MCNP5 are based upon the 8th release of ENDF/B‐VI.^(25)^ ${}^{*}F8$ tally following $10^{9}$ histories and F6 tally following $10^{7}$ histories were used in this study to perform the phantom dosimetry and the air kerma simulations respectively, in order to achieve a relative statistical error of less than 1%.

F6 tally, which estimates the deposited energy equivalent to the collisional kerma, is used to score kerma on terms of MeV/g per photon in the cells. The dose in air can be calculated directly using the F6 tally option of MCNP to calculate the air kerma rate. ${}^{*}F8$ tally is determined based on full charged particle transport physics and it estimates the average deposited energy in units of MeV. The energy absorbed by a given cell must be divided by the mass of the cell in order to achieve the appropriate units: $\text{MeV}/g^{-1}$. It can be modified by a constant multiplier ($1\;\text{Mev}/g=1.602^{*}10^{-10}J/\text{Kg}$) to get dose values in units of Gy.

The current Monte Carlo study was conducted with two phantom test cases (water phantom and complete simulated human eye), in which the ophthalmic brachytherapy dosimetry was evaluated in both cases, in the presence and in the absence of gold nanoparticles using the full loaded eye plaque containing ${}^{125}I$ and ${}^{103}\text{Pd}$ sources. We simulated the human eye globe in a manner similar to that in our previous work.^(21)^

In order to define complete human eye globe, its components have been simulated using interface among different shapes with specific geometry and characteristics. Hence, all the details relating to the simulated phantoms are based upon the mentioned report. Furthermore, in addition to the 16 mm COMS eye plaque loaded with 13 ${}^{125}I$ seeds, a 16 mm eye plaque containing 13 ${}^{103}\text{Pd}$ seeds with the exact same coordinates was defined. The center of the simulated eye globe coincided with the origin.

Rows of cubic voxels with individual volumes of 0.05 cm^3^ were utilized to evaluate the depth dose of the ${}^{125}I$ and ${}^{103}\text{Pd}$ radionuclide sources within both water and eye phantoms. The mentioned voxels were assumed to be arranged on the central axis of the eye. An equator temporal eye melanoma with a height of 0.5 cm is considered in the present study. The COMS‐style eye plaque with the diameter of 16 mm was modeled on the equator temporal periphery to the eyeball.

It is a scientifically proven fact that the optimum GNP uptake within cancerous cells occurs when the noted substances are roughly 50 nm in diameter;^(23)^ hence, the study benefits from the deployment of 50 nm GNPs within the tumor with varying concentrations of 7 mg, 10 mg, 18 mg, and 30 mg. Given the aforementioned fact, the tumor is latticed by identical cubes each with a volume of 0.1 cm^3^.

The dose enhancement factor (DEF) has been calculated to compare the effects of the presence of GNPs in brachytherapy for the noted configurations of sources and phantoms. DEF is the ratio of the absorbed dose by the tumor when it is loaded with GNPs to the absorbed dose by the tumor without these nanoparticles. Also, the dose distribution in different points of the eye has been determined for two mention sources in both eye and water phantoms. Validity of dosimetry computations for both the simulated sources were investigated by parameterized calculations of TG‐43 dosimetry parameters such as air kerma strength, dose rate constant, and radial dose function (RDF), which were then compared with the reported results of Thomson et al. and Rivard et al.^3^, ^26^ For RDF calculations each of the brachytherapy sources were simulated within a water phantom 30 cm^3^ in volume. Furthermore, for the calculations related to dose falloff, toroid tally cells (torus‐shaped cells) with major radii in the range of 0.5 cm to 10 cm were simulated.

## III. RESULTS

### A. Calculations of TG‐43 parameter

**Figure 2:**
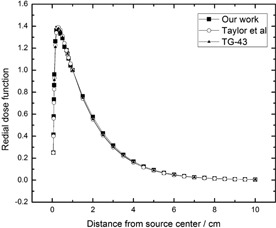
The calculated radial dose function for ${}^{103}\text{Pd}$ source. The relative statistical uncertainties are less than 1%. The size of voxels in which the dose was scored are the same in as the previous work.^(21)^

The calculated RDF in the present work, as reported by Thomson et al.,^(3)^ and by TG‐43^(26)^ are shown in Table 1 and Fig. 2. Also, the calculated dose rate constant in this work, as reported by Thomson et al., and by TG‐43 are shown in Table 2. The results show excellent agreement. It is also noteworthy to mention that all the referred TG‐43 dosimetry parameters were calculated for ${}^{125}I$ in our previous work.^(21)^

**Table 1:** A comparison between the simulated (RDF) of ${}^{103}\text{Pd}$ source in this work with the published data by other investigators.

*Radial Dose Function, g(r)*			
*Distance from source (cm)*	*This work*	*Thomson et al*.^(3)^	*TG‐43* ^(26)^
0.05	0.251	0.249	—–
0.06	0.414	0.401	—–
0.07	0.580	0.561	—–
0.08	0.735	0.704	—–
0.09	0.864	0.826	—–
0.1	0.962	0.929	0.911
0.15	1.263	1.226	1.21
0.2	1.363	1.34	—–
0.25	1.383	1.381	1.37
0.3	1.381	1.39	1.38
0.4	1.332	1.365	1.36
0.5	1.292	1.31	1.3
0.6	1.213	1.245	—–
0.7	1.183	1.18	—–
0.75	1.149	1.148	1.15
0.8	1.106	1.113	—–
0.9	1.042	1.059	—–
1	1.001	1.001	1
1.5	0.763	0.742	0.749
2	0.576	0.552	0.555
2.5	0.426	0.407	0.41
3	0.315	0.298	0.302
3.5	0.231	0.219	0.223
4	0.170	0.16	0.163
4.5	0.124	0.117	—–
5	0.092	0.0865	0.0887
5.5	0.067	0.0635	—–
6	0.050	0.0469	0.0482
6.5	0.037	0.0346	—–
7	0.027	0.0256	0.0262
7.5	0.021	0.0193	—–
8	0.015	0.0147	—–
8.5	0.012	0.0112	—–
9	0.009	0.0084	—–
9.5	0.007	0.0064	—–
10	0.005	0.0051	0.00615

**Table 2:** A comparison of the dose rate constant of ${}^{103}\text{Pd}$ brachytherapy source in water, simulated in this project, with the published data.

	*Taylor et al*.^(27)^	*Rivard et al*.^(26)^	*This Work*
Dose rate constant	0.772	0.686	0.69±0.01

### B. Dosimetry calculations

**Figure 3:**
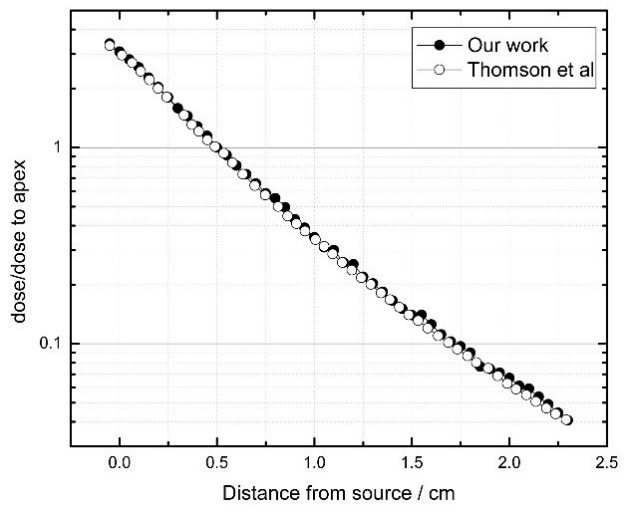
The ratio of plaque central axes to the dose at the tumor apex for ${}^{103}\text{Pd}$ in the water phantom. The results have been compared with Thomson et al.^(3)^

Figure 3 shows the depth‐dose curve in the plaque central axis direction for ${}^{103}\text{Pd}$ source in the fully‐loaded 16 mm COMS eye plaque in water phantom where the dose is presented relative to the dose at the tumor apex. The results have been compared with those reported by Thomson et al.,^(3)^ showing excellent agreement.

The dose to points of interest has been calculated in the water phantom and eye globe for both ${}^{125}I$ and ${}^{103}\text{Pd}$ sources and the results are reported in Table 3. Having yielded a relative statistical uncertainty less than 1% with the maximum percentage being apparent in the opposite side of the eye and the minimal amount of the noted factor observable in the sclera, the study has tabulated the full body of prescription points within the aforementioned table. The results indicate that in a healthy tissue such as the lens the difference in the calculated absorbed dose between water and eye phantoms in varying concentrations of GNPs is around 19% for the ${}^{125}I$ source and 14% for the ${}^{103}\text{Pd}$ source. The deviances for both phantoms in the presence of GNPs are greater when compared to those that were in the absence of the mentioned substance.

**Figure 4:**
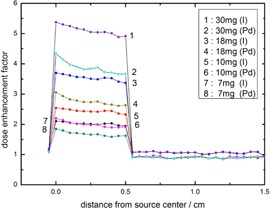
The calculated (DEF) for 50 nm GNPs within the tumor with concentrations of 7, 10, 18, and 30 mg/g of tumor. The calculations have been done in the water phantom with fully loaded 16 mm COMS eye plaque of ${}^{125}I$ and ${}^{103}\text{Pd}$ sources.

**Figure 5:**
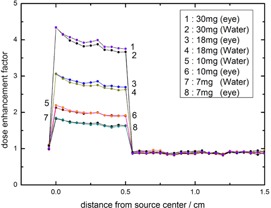
The calculated (DEF) for 50 nm GNPs within the tumor with concentrations of 7, 10, 18, and 30 mg/g of tumor in the eye and water phantoms for ${}^{103}\text{Pd}$ source.

The dose enhancement factor (DEF) has been calculated for both ${}^{125}I$ and ${}^{103}\text{Pd}$ sources. This calculation has been done for different concentration of GNPs within the water phantom and compared with the calculated DEF for ${}^{125}I$ in Fig. 4. As is seen in this figure, the resultant DEF in the apex of the tumor for GNP concentrations of 30, 18, 10, and 7 mg/g are equal to 4.91, 3.37, 2.32, 1.94, respectively, for ${}^{125}I$ source, while being 3.66, 2.63, 1.91, and 1.62, respectively, for the ${}^{103}\text{Pd}$ source. The apparent deviances for the mentioned sources are greater for higher concentrations. A comparison between water phantom and eye globe in calculation of dose enhancement factor for ${}^{103}\text{Pd}$ is shown in Fig. 5. The difference between these phantoms is greater for higher concentrations. For instance, in the concentration of 30 mg GNPs, the calculated difference in the DEF at the apex of the tumor between water phantom and eye phantom is about 2.45%, while the noted deviance is about 0.7% in the concentration of 7 mg GNPs. The tally cells have been placed along the central axis of the plaque, starting from the sclera near the plaque to the sclera opposite side of the plaque. The points located on the transverse plane at distances of 0 to 0.5 cm indicate the center of voxels that were defined within the GNP‐loaded tumor. The points beyond 0.5 cm were in the healthy tissues. The DEF falloffs beyond 0.5 cm in both 4, 5 show the least effects of the presence of GNPs within the tumor on healthy tissues.

**Table 3:** A comparison of the dose ($\text{unit of}\;(\text{Gy}/\text{per particle})\times 10^{-15}$) to the critical points of the eye in the water and eye phantoms for both fully loaded 16 mm (13 seeds) COMS standard ${}^{125}I$ and ${}^{103}\text{Pd}$ eye plaques. Eye refers to the eye phantom and water refers to the water phantom in which the eye phantom was filled of water. 7 mg/g, 10 mg/g, 18 mg/g, and 30 mg/g refer to the concentration of GNPs inside the tumor.

*Water*										
*Location*	${}^{125}I$	${}^{103}Pd$	$7mg/g\;{(}^{125}I)$	$7mg/g\;{(}^{103}Pd)$	$10mg/g\;{(}^{125}I)$	$10mg/g\;{(}^{103}Pd)$	$18mg/g\;{(}^{125}I)$	$18mg/g\;{(}^{103}Pd)$	$30mg/g\;{(}^{125}I)$	$30mg/g\;{(}^{103}Pd)$
Sclera	138.97	217.97	165.45	241.12	165.56	241.00	165.86	235.10	166.16	235.86
Apex	41.03	64.31	79.78	104.18	95.22	123.00	138.29	169.24	201.48	235.67
Center of eye	13.85	19.28	14.09	16.86	14.01	16.80	14.01	16.78	14.10	16.76
Opposite side	2.91	2.62	3.22	2.12	3.20	2.13	3.20	2.37	3.24	2.48
Optic nerve	4.99	5.05	5.07	4.59	5.07	4.59	5.07	4.74	5.08	4.93
Lens	9.54	11.92	10.72	11.41	10.77	11.40	10.77	11.34	10.73	11.39
Macula	6.97	7.68	7.72	7.48	7.71	7.48	7.71	7.52	7.77	7.47
					*Eye*					
Sclera	174.89	263.62	184.90	265.00	185.18	259.47	185.53	265.26	185.81	265.95
Apex	43.12	64.37	80.50	105.00	96.21	122.39	138.46	173.59	200.69	241.61
Center of eye	14.76	18.82	13.46	16.20	13.48	16.25	13.47	16.67	13.53	16.66
Opposite side	3.75	2.89	3.41	2.41	3.39	2.95	3.39	2.69	3.38	2.69
Optic nerve	4.81	4.95	4.96	4.42	4.97	4.48	4.97	4.60	4.96	4.59
Lens	8.55	10.23	8.74	9.71	8.65	9.71	8.65	9.73	8.62	9.73
Macula	7.14	7.24	7.61	7.11	7.64	7.39	7.64	7.41	7.65	7.40

## IV. DISCUSSION

Regarding the radiation dose enhancement properties of GNPs, studies have indicated that in radiotherapy of cancerous cells, the presence of these nanoparticles in the tumor site will locally increase the absorbed dose. Delivering the required tumor dose in a shorter period of time will result in lower doses to the normal tissues. In our previous study,^(21)^ the dose enhancement properties of GNPs on choroidal melanoma for ${}^{125}I$ source were investigated. Given the differences in the properties of ophthalmic brachytherapy sources, the effects of the presence of GNPs in the tumor site in minimizing the damage done to normal tissue for each of these sources can be further investigated and compared. The results of this work have been quick to point out that in brachytherapy of choroidal melanoma, the ${}^{125}I$ source has granted us a greater DEF in the presence of GNPs when compared with the ${}^{103}\text{Pd}$ source. It is also noteworthy that the dosimetry calculations for the eye in the presence of GNPs report no notable differences for the absorbed dose in the healthy tissue in comparison with the cases where GNPs were absent for each of the noted sources. In reference to the noted evidence in brachytherapy of eye melanoma in presence of these particles, it is more efficient to utilize the ${}^{125}I$ source for it grants a higher DEF when compared with the ${}^{103}\text{Pd}$ source. The importance of defining an actual eye phantom instead of the water phantom in Monte Carlo studies has been another factor gaining much attention in recent years. The results signify that calculations of the resultant DEF in the tumor for the brachytherapy of eye melanoma utilizing a ${}^{103}\text{Pd}$ source show no striking difference when the actual eye model and the water phantom are compared. This accords with the previous study,^(21)^ where the results pointed out that the use of the actual eye phantom in the Monte Carlo study of the dosimetry in eye melanoma with ${}^{125}I$ source, whilst GNPs were present, was of utmost importance. Furthermore, the results indicate that the deviances for both water and eye phantoms in the presence of GNPs are greater when compared to those that were in the absence of the mentioned substance. Regarding the numerous sources that are employed in the radiotherapy of the eye melanoma, the possible effects of these sources when accompanied by GNPs, regarding altering the period of treatment, is a matter of heated debate and requires more comprehensive investigations.

## V. CONCLUSIONS

This work indicates that the presence of GNPs as a method of dose enhancement in treatment of eye tumors grants a higher DEF for the ${}^{125}I$ brachytherapy source as opposed to that of the ${}^{103}\text{Pd}$ source. Previous studies have been quick to point out that in the period of brachytherapy treatment of the eye (in the absence of GNPs), the ${}^{103}\text{Pd}$ source generates a greater dose in the tumor site. Since the results designate that the difference between absorbed dose in normal tissue, and in the presence of GNPs, is negligible when the two sources are compared, it is safe to say that the use of the ${}^{125}I$ source along with GNPs would yield a higher DEF in the tumor site of the eye melanoma. The previous study has also specified that ever‐more‐accurate definition of the actual eye phantom, instead of the water phantom, is an absolute necessity for precise dosimetry of the eye melanoma when examination utilizing the ${}^{125}I$ source is intended. However, even so, the dosimetry results obtained for the ${}^{103}\text{Pd}$ source in both the water and the eye phantoms are roughly similar; thus, in brachytherapy studies of the eye involving Monte Carlo methods, designation of a water phantom as an alternative for the eye phantom causes no observable difference in the results, and efficiently replicates those of the eye phantom.

## COPYRIGHT

This work is licensed under a Creative Commons Attribution 4.0 International License.
